# Biomedical Applications of Non-Small Cell Lung Cancer Spheroids

**DOI:** 10.3389/fonc.2021.791069

**Published:** 2021-12-07

**Authors:** Julian M. Rozenberg, Gleb I. Filkov, Alexander V. Trofimenko, Evgeny A. Karpulevich, Vladimir D. Parshin, Valery V. Royuk, Marina I. Sekacheva, Mikhail O. Durymanov

**Affiliations:** ^1^ Cell Signaling Regulation Laboratory, Moscow Institute of Physics and Technology (National Research University), Dolgoprudny, Russia; ^2^ Laboratory of Medical Informatics, Yaroslav-the-Wise Novgorod State University, Veliky Novgorod, Russia; ^3^ Special Cell Technology Laboratory, Moscow Institute of Physics and Technology (National Research University), Dolgoprudny, Russia; ^4^ Department of Information Systems, Ivannikov Institute for System Programming of the Russian Academy of Sciences, Moscow, Russia; ^5^ Clinical Center, Sechenov First Moscow State Medical University, Moscow, Russia; ^6^ World-Class Research Center “Digital Biodesign and Personalized Healthcare”, Sechenov First Moscow State Medical University, Moscow, Russia

**Keywords:** spheroid model, non-small cell lung cancer, drug screening, personalized medicine, immunotherapy

## Abstract

Lung malignancies accounted for 11% of cancers worldwide in 2020 and remained the leading cause of cancer deaths. About 80% of lung cancers belong to non-small cell lung cancer (NSCLC), which is characterized by extremely high clonal and morphological heterogeneity of tumors and development of multidrug resistance. The improvement of current therapeutic strategies includes several directions. First, increasing knowledge in cancer biology results in better understanding of the mechanisms underlying malignant transformation, alterations in signal transduction, and crosstalk between cancer cells and the tumor microenvironment, including immune cells. In turn, it leads to the discovery of important molecular targets in cancer development, which might be affected pharmaceutically. The second direction focuses on the screening of novel drug candidates, synthetic or from natural sources. Finally, “personalization” of a therapeutic strategy enables maximal damage to the tumor of a patient. The personalization of treatment can be based on the drug screening performed using patient-derived tumor xenografts or *in vitro* patient-derived cell models. 3D multicellular cancer spheroids, generated from cancer cell lines or tumor-isolated cells, seem to be a helpful tool for the improvement of current NSCLC therapies. Spheroids are used as a tumor-mimicking *in vitro* model for screening of novel drugs, analysis of intercellular interactions, and oncogenic cell signaling. Moreover, several studies with tumor-derived spheroids suggest this model for the choice of “personalized” therapy. Here we aim to give an overview of the different applications of NSCLC spheroids and discuss the potential contribution of the spheroid model to the development of anticancer strategies.

## Introduction

**Figure 1:**
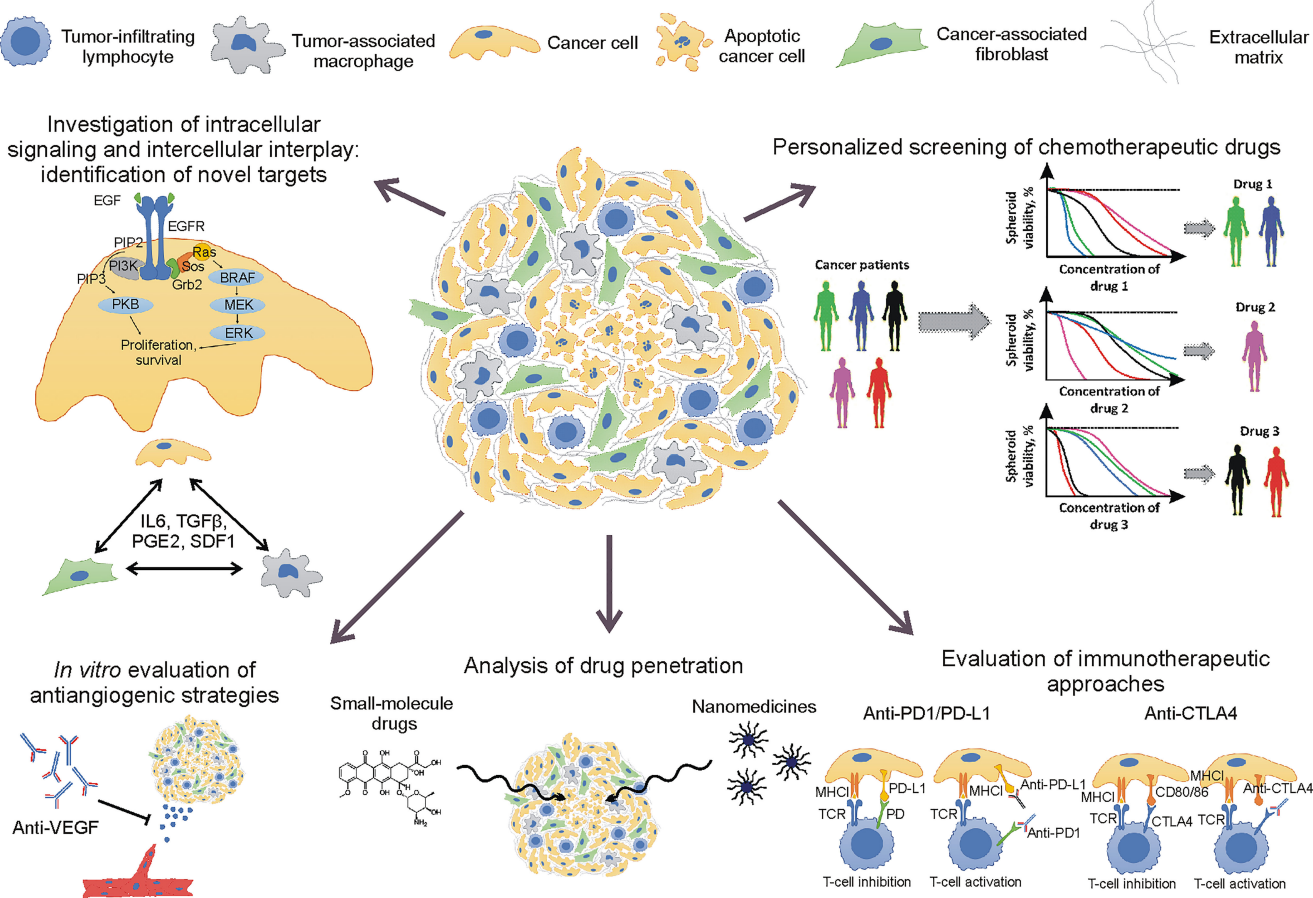
Application of non-small cell lung cancer spheroids for cancer biology and drug delivery studies.

Non-small cell lung cancer (NSCLC) with 25% 5-year survival rate (1) remains an intractable type of cancer. One of the challenges for NSCLC treatment is an evolution of cancer genomes that leads to clonal heterogeneity, recurrent mutation occurrence, and, therefore, development of drug resistance and therapeutic failure (2, 3). Other NSCLC hallmarks, such as immune tolerance (4), high level of extracellular matrix (ECM) content (5), and hypoxia (6), also complicate cancer therapy and correlate with poor prognosis. It means that the reproduction of the mentioned hallmarks in tumor models is important for the improvement of current therapies and the development of novel strategies. For this reason, multicellular 3D spheroid models are becoming an important tool for cancer biology and drug delivery studies (**Figure 1**).

## Key Features of NSCLC Spheroid Model Relevant to Patient Tumors

A spheroid model has been shown to have some features relevant to patient NSCLC tumors. The properties of the obtained spheroids strongly depend on the cell composition and culture technique. The simplest NSCLC multicellular spheroids can be generated from one cell line, resulting in a homotypic model. More complex heterotypic spheroid models, comprising two or more cell types, are generally used for studying of the interplay between cancer cells and other cell types, such as immune and stromal cells. For the best mimicking of the tumor microenvironment and “personalized” drug testing, patient-derived (also called “organotypic”) spheroid models were introduced. There are multiple methods of spheroid generation. The choice of spheroid culture technique is determined by the goal of the study—for example, scaffold-based techniques can be used for the analysis of cancer cell invasiveness (7). Scaffold-free techniques, including cell centrifugation, placing the cells into hanging drop, rotation of the vessel with cells, and cultivation on non-adhesive surfaces, are more frequently used for drug testing, analysis of intercellular interactions, and cell signaling studies. Multiple parameters of the obtained spheroids, such as mean diameter, size heterogeneity, number of spheroids, and duration of cultivation, are determined by the method of spheroid generation (8). All these parameters should be taken into account during the design of the experiment.

### Cell–Cell Contacts

Spheroid formation using scaffold-free techniques encourage cell–cell interactions primarily mediated by cadherins (9). E-cadherins and N-cadherins are two main members of calcium-dependent cell adhesion molecules involved in the formation of adherens junctions. The molecular ratio between E- and N-cadherins in lung cancer cells strongly affects their migratory properties. As a part of the epithelial–mesenchymal transition (EMT), the prevalence of N-cadherins significantly contributes to RhoA signaling activation that mediates cell locomotion and invasiveness (10) and is associated with poor prognosis in NSCLC (11). Both types of cadherins participate in cell–cell interaction during spheroid formation that has been demonstrated using homotypic spheroid models composed of only one cancer cell line. In particular, generation of spheroids from NSCLC cell lines A549 and H1299 resulted in one-order upregulation in N-cadherin level and a decrease in E-cadherin expression compared with those cell lines in 2D cultures. Moreover, the cells in spheroids demonstrated an enhanced expression of anti-apoptotic Bcl-2 and chemoresistance markers MDR1 and ABCG2 (12).

### Hypoxic Core Formation

Cell aggregation and further growth lead to the appearance of a hypoxic core in the central area of a spheroid. Hypoxia is an important hallmark of lung tumors, which affects cancer cell metabolism and chemoresistance (13). Transcriptional factor HIF1α acts as a master regulator of numerous hypoxia-responsive genes. HIF1α causes a shift of cancer cell metabolism to glycolic pathway by upregulation of glucose transporters GLUT1 and 3, glycolytic enzymes (HK, ALDO, ENO, *etc.*), and enzymes involved in lactate production and lactate/proton elimination (LDH-A, MCT4, and CA9) (14). Thus, HIF1α activation enables cancer cells to generate ATP in anaerobic conditions and to regulate the intracellular pH level. It has been shown that avoiding intracellular acidosis significantly contributes to the chemoresistance of lung adenocarcinoma A549 cells due to the overexpression of carbonic anhydrase 9 (СА9) (15), activated by hypoxia-driven HIF1α and EMT-inducing ZEB1 transcriptional factors (16, 17). Once chemotherapy induces intracellular acidosis and apoptosis, a high level of CA9 maintains the intracellular pH and prevents cell death (17). In addition, CA9 induces extracellular acidosis by converting CO_2_ and H_2_O into H^+^ and HCO_3_^−^ in the extracellular space that leads to protonation of anticancer drugs and impairment of their efficacy (18). Besides chemoresistance, hypoxia contributes to the metastasis of NSCLC *via* upregulation of metalloproteinases (MMPs) and lysyl oxidase (LOX) involved in ECM remodeling and cancer cell migration (19). Furthermore, HIF1α induces VEGF expression and angiogenesis, which negatively correlate with survival in stage IIIA NSCLC (20). Although anti-angiogenic therapy demonstrated mixed results in clinical trials of advanced lung cancer, it is still considered a promising strategy (21). Hypoxia was detected in NSCLC spheroids (22), suggesting a similarity in gene expression alterations in patient tumors and spheroids. It has been shown that A549-based spheroids display a threefold higher production of pro-angiogenic VEGF-A and bFGF compared with cells in monolayer (23).

### Extracellular Matrix Deposition

Besides hypoxic core formation, the growth of the spheroids leads to the deposition of extracellular matrix (ECM) components, which is an important hallmark of NSCLC tumors. It is thought that the main contribution to ECM production belongs to cancer-associated fibroblasts (CAFs). Cancer cells secrete TGFβ1 and chemokines, which activate resident fibroblasts and induce their differentiation into CAFs (24). CAFs participate in ECM remodeling *via* overexpression of MMPs 2 and 9, LOX, collagen type I, fibronectin, and tenascin-C (25). The remodeled ECM may trigger the migratory and proliferative potential of lung cancer cells, mainly due to integrin receptor signaling (26). Moreover, excessive ECM deposition impairs drug penetration in tumor tissue (27). For better mimicking of the tumor ECM, heterotypic spheroid models have been developed. Introduction of fibroblasts into A549 spheroids resulted in a marked increase in collagen I, laminin, and fibronectin expression in heterotypic spheroids in comparison with homotypic counterparts (28).

### Immunosuppressive Microenvironment

Heterotypic spheroid models are often used for mimicking the immune microenvironment. NSCLC tumors are characterized by an immunosuppressive microenvironment generated due to multiple and interconnected mechanisms, including HLA-G expression by cancer cells (29), activation of inhibitory immune checkpoint mechanisms (30), release of anti-inflammatory cytokines and mediators (31), indoleamine 2,3-dioxygenase overexpression (32), Treg activation (33), and M2 macrophage polarization (34). It has been shown that a 3D co-culture of NCI-H157 lung cancer cells and lung-derived CAFs promoted the M2 polarization of THP-1 monocytes within a heterotypic spheroid. These spheroids also displayed an elevated expression of IL-4, CCL22, CCL24, and MMPs compared with monocultures (35). Another spheroid model, consisting of H522 lung cancer cells and AG02603 fibroblasts, stimulated the acquisition of an immunoregulatory M2-like phenotype by peripheral blood mononuclear cells (36). It should be noted that patient tumors contain a broader spectrum of cell types than heterotypic spheroids. For this reason, patient-derived spheroid models were introduced for the study of the immune microenvironment of tumors and testing immunotherapeutics (37).

### Clonal Heterogeneity

Another hallmark of patient tumors, which can be reproduced in organotypic spheroids, is clonal heterogeneity. NSCLC tumors are highly heterogeneous from the genetic point of view that results in a non-uniform response to therapies, mutational evolution, and development of drug resistance (3). Clonal heterogeneity has been recently reported for NSCLC patient-derived multicellular 3D cultures (38).

### Expression of Pluripotency Markers

Cancer stem cells contribute to the development of drug resistance and cancer relapse. Therefore, development of therapeutics aiming at the eradication of cancer stem cells is crucial for cancer treatment.

In comparison with adherent cultures, spheroids contain more cells expressing “stemness” markers, including c-Myc, Sox2, OCT4, Nanog, KLF4, CD133, CD44, and β-catenin (39–42). At the same time, spheroids are typically less proliferative and less sensitive to cytotoxic drugs than adherent cultures. In this regard, the pluripotency markers in spheroids are often evaluated in parallel with drug testing and tumor forming ability in mice (43–47).

Many studies suggest that “stemness” correlates directly with tumor formation and aggressiveness—for example, an evaluation of gene expression in spheroids and adherent cultures from NSCLC cell lines and primary cell cultures revealed the overexpression of n-Myc downstream regulated gene 1 (NDRG1) that, in turn, stabilizes c-Myc, maintaining other pluripotency markers, and is associated with advanced disease (40). Similarly, spheroids generated from primary cells are more resistant to chemotherapy and display higher tumorigenicity than adherent cell cultures. Compared to cells in monolayers, spheroids demonstrated a higher expression of stem cell marker genes *NANOG*, *CD44*, *NOTCH3*, *CDKN1A*, *SNAI1*, and *ITGA6*, the level of which correlates with shorter survival of the NSCLC cohort from The Cancer Genome Atlas (48).

Thus, the spheroid model mimics multiple features of the patient tumors. In this context, a spheroid model is considered a reliable tool for different studies, including analysis of molecular crosstalk in tumor microenvironment, identification of cell–cell interactions, and drug screening.

## Study of Cancer Cell Signaling in NSCLC Spheroids

Signaling pathways, induced by growth factor receptors, play a pivotal role in tumor growth and development of multidrug resistance. The excessive signal transduction in NSCLC tumors originates from activating mutations in oncogenes, including *KRAS* (30%), *EGFR* (19%), *BRAF* (5%), *HER2* (3%), *MET* (3%), and *ALK* (3%) (49).

Epidermal growth factor receptor (EGFR) tyrosine kinase (TK) inhibitors have been used to treat NSCLC, although resistance to them typically develops after 10 months. It was shown using the spheroid model that the acquisition of resistance to EGFR inhibitor gefitinib is mediated by the induction of transcription factor Sox9, which contributes to β-catenin overexpression and EMT (50). Another study reports that treatment of patient-derived NSCLC spheroids with erlotinib leads to a change in clonal composition of the spheroids, resulting in the outgrowth of erlotinib-resistant subpopulations (38). Gefitinib and erlotinib, referred to as first-generation EGFR TK inhibitors, are able to interact with the ATP-binding pocket in the TK domain in a reversible manner, whereas second-generation EGFR TK inhibitors afatinib and dacomitinib bind irreversibly. It was found that most of the patients develop resistance to first- and second-generation EGFR TK inhibitors *via* acquisition of the additional mutation T790M in the ATP-binding pocket. This mutation leads to a higher binding affinity of the ATP-binding pocket to ATP than to TK inhibitory molecules (51). The development of a third-generation drug, osimertinib, enabled T790M-mediated resistance to be overcome and caused superior therapeutic outcome in this cohort of patients compared with platinum-based therapy plus pemetrexed (52). However, tumors can also develop resistance to osimertinib due to second site mutations in EGFR or the activation of bypass pathways (51). The use of a patient-derived spheroid model would help to determine whether combinations of different EGFR TK inhibitors can prevent the appearance of the resistant clones.

To bypass the resistance to EGFR TK inhibitors, multiple participants in the signal transduction pathway can be targeted simultaneously—for example, combined EGFR and SOS1 inhibition repressed Raf/MEK/ERK and PI3K/Akt signaling and synergized in the inhibition of spheroid growth but not of adherent cultures. A detailed analysis of the inhibition of the molecules, acting downstream of EGFR, showed that the synergistic effect is achieved for molecules upstream of RAS, whereas inhibition of molecules downstream of RAS failed to produce synergy (53). RAS itself is also a highly attractive molecular target because mutations of the *RAS* gene family are the main driving force of numerous cancers. Recently, KRAS inhibitor AMG510 was developed by Amgen and approved by the FDA for NSCLC patients who have KRAS G12C mutation and who have been previously treated. During the development of AMG510, NCI-H1373, NCI-H2122, and NCI-H358, lung adenocarcinoma cell lines were tested in both adherent cultures and spheroids which, in contrast to chemotherapeutics, had higher sensitivity to AMG510. In addition, the combinational treatment of cell lines with inhibitors that target protein kinases located upstream of RAS in MAPK signaling pathway revealed an enhancement of efficiency both in adherent cultures and spheroids. In addition, simultaneous treatment with PD-1 and AMG510 demonstrated synergistic effects in mice models. A preliminary trial in patients with advanced lung cancer showed promising results (54), and clinical trials are underway.

NSCLC spheroids were also used for the evaluation of potential inhibitors of the PI3K/Akt pathway, another branch of EGFR signaling. It was revealed that aspirin represses mTOR gene transcription, which led to the reduction of Akt phosphorylation, GSK3β activation, and Snail and β-catenin destabilization, resulting in enhanced cisplatin sensitivity of NSCLC spheroids (46). Melatonin treatment inhibited spheroid formation by H460 cells due to a decrease of Akt phosphorylation, EMT markers, CD133, Oct-4, Nanog, and β-catenin. Interestingly, this effect was not mediated by the melatonin receptor, prompting investigations of the alternative mechanisms (55).

Apparently, to increase the effectiveness of an anticancer treatment, multi-targeted approaches have to be developed. Specifically, the mechanisms that maintain immunosuppression and drug resistance in cancers rely on the interaction of multiple cell types readily investigated in spheroids.

## Study of Intercellular Crosstalk in NSCLC Spheroids

Both artificial heterotypic and patient-derived spheroids contain cell types typically found in a cancer microenvironment. Thus, spheroid models are used to investigate how drugs or biologicals, such as natural chemokines or cytokines, affect intercellular communications (35, 36, 56).

### Cancer Cell/Stromal Cell Interactions

Stromal cells including cancer-associated fibroblasts and mesenchymal stromal cells (MSCs) promote immunosuppression due to the production of TGFβ and IL6 (57, 58), induce the EMT of cancer cells, and support cancer stem cell proliferation and chemoresistance (58). It has been shown that addition of fibroblasts renders A549 spheroids with additional resistance to EGFR inhibition (56), likely by IL6 secretion that promotes STAT3-mediated cell survival (59–61).

A co-culture of primary squamous lung carcinoma cells with MSCs led to the secretion of CCL3 by cancer cells and an elevated expression of IL-6, CCL2, ICAM-1, and VCAM by MSCs. These changes were suppressed by the lipid-lowering drug simvastatin, which suppressed IL-6 and CCL2 production by MSCs, inhibiting spheroid formation and the survival of cancer cells (62).

Thus, several investigations point at a few signaling molecules/signaling pathways involved in the NSCLC stromal cancer cell crosstalk, including IL6, TGFβ, and CCL2.

### Cancer Cell/Endothelial Cell Interaction

Interaction between lung cancer cells and endothelial cells promotes radio- and chemoresistance by inducing the EMT in lung cancer cells (63).

It was found that a co-culture of HUVECs with NCI-H460 or A549 cells in spheroids or medium conditioned by co-culture induces the resistance of lung cancer cells to cisplatin and EGFR inhibitor gefitinib. Using microarray analysis and functional assays, it was determined that the Hsp70 family protein HYOU1 plays a key role in resistance as well as cell viability, levels of EMT markers, stem cell marker CD133, and interferon signaling in the spheroids. Treatment of cells with PI3K or mTOR inhibitors decreased the HYOU1 level, suggesting the regulation of HYOU1 by the PI3K/Akt/mTOR signaling pathway (64). Altogether these define HYOU1 as a new therapeutic target for NSCLC.

In turn, lung cancer spheroid secretome induces EMT in HUVECs, while adhesive cultures are not. Interestingly, the expression of GSK-3β-targeted genes was altered in multicellular spheroids, and the inhibition of GSK-3β induced EMT reversion (65). In addition, the inhibition of GSK-3β decreased the volume of lung cancer and synergized with gefitinib in the xenograft model.

Thus, spheroid models of interacting lung cancer and endothelial cells provide valuable information for the identification of novel targets for lung cancer treatment.

### Cancer Cell/Immune Cell Interaction

As mentioned earlier, NSCLC tumors are characterized by an immunosuppressive microenvironment, and spheroids reproduce this property.

Cytotoxic T-cells play a pivotal role in anticancer immunity, and T-cell infiltration determines a favorable prognosis for many cancers as well as sensitivity to inhibitors of immune checkpoint receptors, including PD-L1, PD-1, and CTLA-4. Besides immune checkpoint receptor expression, avoiding T-cell-mediated immunosurveillance occurs due to HLA downregulation in cancer cells as shown using IGR-Heu lung large cell carcinoma-based spheroids (66). Interestingly, in the NSCLC cell lines, MEK-1 inhibition reduced the level of PD-L1 and induced MHC-I expression and proinflammatory cytokines. In turn, the combinational treatment of patient-derived spheroids with MEK inhibitor and PD-L1 antibodies has a stronger effect on viability than each treatment alone (67). Thus, combinations of MEK and immune checkpoint inhibitors seem to be a promising approach and currently are tested in clinical trials for NSCLC (68).

Among the cells of innate immunity, natural killer (NK) cells demonstrate a major anticancer effect by recognizing the cells with decreased MHC-I expression. To investigate NK cell migration and cytotoxicity against A549 spheroids, a simple transwell system was used. It was found that elevation of PGE2 concentration attenuates the NK cell migratory capacity towards spheroids (69). Thus, the obtained data contributed to the understanding of anticancer effects of COX-2 inhibitors. Different adjuvants can also be used for the induction of IFN signaling and NK cell killing activity—for example, infection by oncolytic parainfluenza virus enhanced the NK-mediated death of spheroids from lung cancer cell lines. Although only the outer cell layer in the spheroids was infected by the virus, it was enough for the NK-mediated killing of interior uninfected cancer cells (70).

In contrast to T-cells and NK cells, neutrophil infiltration promotes the growth of the A549 spheroids. It was found that the recruitment of neutrophils occurred in a CXCR2-dependent manner. Treatment with CXCR2-specific antagonist strongly inhibited A549 spheroid growth (71).

Thus, investigation of the cancer cell–immune cell interplay using spheroid models might help to discover novel therapeutic targets or to understand the molecular mechanisms of current immunotherapeutic approaches.

## NSCLC Spheroids for Predictive Screening of Currently Used Chemotherapeutic Drugs

Neoadjuvant and adjuvant chemotherapies are commonly adopted therapeutic strategies for the treatment of stages II and III NSCLC tumors before or after surgical resection, respectively (72). There are two most commonly used groups of drug combinations. The first combination includes a platinum derivative (cisplatin or carboplatin) with a drug of different action mechanism, including Vinca alkaloid derivatives, taxanes, etoposide, mitomycin, or others (73). The second combination includes gemcitabine, usually with paclitaxel (74) or other drugs. In addition, targeted therapy drugs can be prescribed. Thus, several drug combinations can be potentially used. According to meta-analysis data, all these combinations demonstrated similar efficacies with a 5% increase of the 5-year survival rate (75). It is believed that “personalization” of adjuvant chemotherapy would be beneficial for the patients. Personalized strategy implies determination of an anticancer drug or drug combination which causes the best therapeutic response in a certain tumor. Organotypic spheroids, generated from surgically resected material or biopsy, seem to be a promising model for prognostic drug screening.

## Chemoresistance of NSCLC 3D Spheroids

Compared with 2D cancer cell cultures, spheroids are more resistant to cytotoxic drugs. The spheroids, based on NSCLC cell line Colo699, displayed up to 10-fold higher resistance to cisplatin and vinorelbine in comparison with the cells in a monolayer (76). Similarly, enhanced resistance to etoposide, camptothecin, and doxorubicin was demonstrated by the spheroids, composed of INER-37 and INER-51 cell lines, as opposed to their 2D counterparts (77). The chemoresistance of 3D cultures can be explained by multiple causes. First, the drug penetration of the spheroids is limited (78) due to the high cell density and barrier function of the ECM (79). Hypoxic conditions, visualized in a A549-based spheroid core (22), might also contribute to drug resistance, as was mentioned above. Cell–cell interactions between cancer cells are also important for the acquisition of chemoresistance. It was shown that claudin-2, involved in tight junction contact formation, is highly expressed in A549 cells. The downregulation of claudin-2 in A549 cells increased the paracellular permeability and doxorubicin accumulation in A549-based spheroids. It was also found that claudin-2 positively regulates the expression of multidrug resistance-associated protein ABCC2. As a result, the sensitivity of A549 spheroids with reduced claudin-2 level to doxorubicin was significantly increased (80).

Additionally, other cell types in patient-derived spheroids can support the proliferation and chemoresistance of cancer cells *via* cytokine production. It has been shown that NSCLC spheroids contain cancer cells, M2-polarized macrophages, fibroblasts, and CD8+ and CD4+ T-cells, and the spheroid cell composition correlates with the original NSCLC tumors (81). Macrophages and cancer-associated fibroblasts, as parts of the spheroid, are able to release IL-6 and TGFβ, which contribute to the resistance of lung cancer cells to ionizing radiation and cytotoxic drugs (58). Thus, patient tumors demonstrate much more similarity of response to chemotherapeutic drugs with the spheroids compared with 2D cultures.

## Patient-Derived NSCLC 3D Spheroids for “Personalized” Choice of Chemotherapy

There are several studies demonstrating the feasibility of patient-derived spheroid use for chemotherapeutic drug screening. It was shown that organotypic NSCLC spheroids can be cultivated up to 120 days, express adenocarcinoma marker, and demonstrate a similarity of response to cisplatin, with spheroids composed of H1299 cells (82). In another study, two patient-derived tumor xenografts (PDX) with mutant EGFR were used as a source of cells for spheroid generation. These spheroids were used for the screening of different neratinib-based drug combinations. In parallel, the same drug combinations were used for the targeted therapy of corresponding PDX-bearing mice. It turned out that the neratinib/trastuzumab combination caused a more robust inhibition of both NSCLC spheroids and PDX tumors. Thus, this study indicated the consistency of targeted therapy drug screening in spheroids and PDX animal model (83). A correspondence between chemo/immunotherapy response in patient-derived spheroids and patient tumor was shown in a case report study. Cisplatin/vinorelbine treatment, followed by anti-PD-1 therapy, resulted in a decrease of 2-deoxy-2-[fluorine-18] fluoro-D-glucose by a tumor as measured by positron emission tomography. Organotypic spheroids, obtained from this patient, also demonstrated high sensitivity to these pharmaceuticals (84), suggesting the consistency of 3D cell culture drug sensitivity with the clinical response of the patient to chemotherapy and first-line immune therapy.

## Prognostic Screening of Currently Used Immunotherapies Using Patient-Derived NSCLC Spheroids

Recently, immune checkpoint inhibitors were approved as a standard of care for first-line therapy in stage IV NSCLC, including anti-PD1 (nivolumab, pembrolizumab, and cemiplimab), anti-PD-L1 (atezolizumab and durvalumab), and anti-CTLA4 (ipilimumab) (85). A significant increase of 5-year survival, up to 10% for stage IV NSCLC, is observed with the application of nivolumab. For patients with stage III NSCLC, pembrolizumab or cemiplimab can be given as the first line of treatment if surgery or chemotherapy with radiation cannot be performed. Apparently, the disease is still lethal for the majority of patients, thus demanding further research (86).

Patient-derived NSCLC spheroids have been used as a possible model to predict tumor sensitivity to immune checkpoint treatment in several studies. All of them suggest that the anti-cancer effect of PD1, PD-L1, or CTLA4 blockade was mediated by autologous, tumor-infiltrating immune cells within the spheroids. These studies revealed a marked increase in pro-inflammatory cytokine expression and CD8+ T-cell fraction (37, 87). However, the predictive value of these models remains unclear because no correlation analysis with outcome for patients has been carried out. A study where the consistency of a therapeutic effect of PD-1 blockade in a patient and organotypic NSCLC spheroids response has been shown was implemented in a single patient (84) and cannot be considered a solid validation of the spheroid model for prognostic screening. Probably, new studies in this area will allow us to realize the prognostic value of patient-derived spheroids for the evaluation of immune checkpoint blockade.

## Analysis of Anti-angiogenic Strategies Using NSCLC Spheroids

Anti-angiogenic therapy aims to abrogate the growth of new blood vessels in tumor tissue and reduce the oxygen and nutrient supply of the tumor. Anti-angiogenic therapy is still considered a promising strategy despite multiple clinical trials which exhibited contradictory results in advanced lung cancer (21). The lack of therapeutic effect in some trials could probably be due to the absence of predictive markers, which would help to define the patients that would benefit most from an anti-angiogenic therapy (88).

In a study by Amann et al. the authors generated homo- and heterotypic spheroids consisting of A549 cancer cells and a co-culture of A549 with SV80 fibroblasts, respectively. After 5 days, endothelial cells were added to both 3D cultures in the presence or absence of anti-angiogenic drugs, such as bevacizumab, an anti-VEGF-A antibody, and nindetanib, an inhibitor of pro-angiogenic receptor-coupled tyrosine kinases (VEGFR, bFGFR, and PDGFR). It was shown that anti-angiogenic drugs reduced the endotheliocyte infiltration of homospheroids in a concentration-dependent manner. At the same time, no significant inhibition was achieved in the case of A549/SV80 co-culture in spite of a much higher expression of bFGF and VEGF-A in these spheroids in comparison with homospheroids (23). These data are consistent with the results of clinical trials, where anti-angiogenic therapy, when used alone, did not improve the therapeutic outcome. In contrast, the combination of anti-angiogenic therapy with chemotherapy resulted in beneficial effects for NSCLC patients due to the temporary “normalization” of the tumor vasculature, which improves blood perfusion and drug uptake by a tumor (89).

## NSCLC Spheroids for the Evaluation of Novel Drug Candidates and Nanomedicines

NSCLC spheroids are widely used for the evaluation of novel drug candidates. To date, there are three main groups of potential therapeutics. The first group includes antibodies against receptors and synthetic inhibitors of enzymes, which are involved in pro-tumorigenic signaling or favorably encourage a tumor growth microenvironment. Another group comprises nanoformulations of different nature, which could be advantageous for NSCLC treatment. The third group includes natural products, which remain an important source of potential drug candidates.

### Evaluation of Novel Drug Candidates for Targeted Therapy

The spheroid model is a valuable tool of precision medicine due to its ability to recapitulate tumors. As discussed earlier, spheroids are used for studies of signaling pathways in cancer cells and in the analysis of interactions between cancer cells and other cell types in a tumor microenvironment. Besides well-known molecular targets involved in cancer cell division, growth, and spread, some others were identified. Moreover, novel inhibitors of cancer signaling were studied recently as potential drug candidates using NSCLC spheroids. **Table 1** shows a summary of the results of selected studies on the evaluation of these molecules, as discussed above.

**Table 1:** Evaluation of novel drug candidates for targeted therapy using a non-small cell lung cancer spheroid model.

Molecular target	Inhibitor	Spheroid model	Anticancer effect	Reference
MCL-1	S63845	*Ex vivo* mice pulmospheres	Genetic deletion impedes pulmosphere growth	(90)
Glut1 and PDH	Bay-876 and CPI-613	H1299 and H1792 homotypic spheroids	Inhibition of spheroid growth	(91)
GSK-3	CHIR-99021	Heterotypic spheroids of lung cancer cell lines (NCI-H460, A549, and SK-MES-1) and stromal cells (WI38 and HUVEC)	Enhances the efficacy of anticancer drugs *in vitro* and *in vivo*	(65)
STAT3	Biscoumarin OT52	Homotypic A549, H460, H1650 spheroids	Inhibition of spheroid formation	(92)
ATP synthase	Oligomycin	H446 spheroids	Inhibition of spheroid formation	(93)
miR-149-5p/MyD88	Ursolic acid	A549 paclitaxel resistant spheroids	Reduced stemness and paclitaxel resistance	(45)
TRAIL	Adenovirus ZD55-TRAIL	A549 spheroids	Induction of A549 cell apoptosis in spheroids	(94)
GO-203	MUC1-C	A549 spheroids	Inhibition of A549 spheroid formation	(95)
Wnt/β-catenin	Trifluoperazine	Patient-derived cell line spheroids	Inhibition of spheroid formation	(96)

### Evaluation of Natural Products as Drug Candidates Using NSCLC Spheroids

Despite some challenges, screening of novel drug candidates from natural sources remains an important anticancer drug discovery approach (97). To date, the list of anticancer drugs for NSCLC treatment includes several phytochemicals and their derivatives, such as taxanes, *Vinca* alkaloids, etoposide, and others. Cancer spheroids seem to be an attractive model for the screening of newly derived natural products because of their enhanced chemoresistance as mentioned above.

Recently, the naturally derived phytochemical plumbagin was evaluated using A549 spheroids. This agent promoted oxidative stress in many cancer cell lines and exhibited pro-apoptotic and anti-proliferative effects. It was shown that plumbagin caused a concentration-dependent shrinkage of spheroids despite their much higher chemoresistance in comparison with A549 cells in monolayer (98). The same spheroid model was used for the evaluation of salinomycin, an antibiotic potassium ionophore. This antibiotic reduced the expression of stem cell markers (OCT-4, Nanog, and SOX2) and caused A549 spheroid rupture (99). Ilhan-Ayisigi et al. synthesized niosome nanovesicles doped with a galangin-rich propolis extract. These nanoparticles demonstrated a profound cytotoxic effect on the A549 spheroid model (100).

The underlying mechanisms of natural products can involve the inhibition of pro-tumorigenic signaling pathways such as Wnt/β-catenin. It was shown that benzophenanthridine alkaloid, called chelerythrine chloride, significantly affects nuclear localization and the overall cellular content of β-catenin. As far as β-catenin participates in adherens junction formation, the treatment of NSCLC spheroids, composed of NCI-H1703 cells, led to their decomposition (101). The authors of this study also evaluated some alkaloids, chalcones, and isothiocyanates for their inhibitory activity to the nuclear localization of β-catenin. It was found that phenethyl isothiocyanate caused the strongest inhibition of β-catenin nuclear accumulation and NCI-H1703 spheroid growth (101). Probably, some of the mentioned natural products will be translated for clinical use in the future.

### Evaluation of Nanomedicines Using NSCLC Spheroid Model

Despite high expectations, nanomedicines demonstrated limited success in clinical translation so far (102). However, some of them were accepted and are used for the treatment of some types of cancer (103). The rational design of nanocarriers or additional co-treatments enable the improvement of their efficacy.

Although there are no clinically accepted nanomedicines for NSCLC treatment, numerous studies investigate the therapeutic effects and other drug delivery aspects of nanoformulations using lung cancer spheroid models. As this topic has been reviewed in detail earlier (79), we provide here a brief description of the selected studies where nanoparticles of different carrier nature and of different therapeutic payloads were tested using NSCLC spheroids (**Table 2**). The anticancer effects of these nanomedicines are also summarized in **Table 2**.

**Table 2:** Non-small cell lung cancer spheroid model for the analysis of the therapeutic effects of nanomedicines.

Nanoformulation	Spheroid model	Anticancer effect	Reference
Spherical nanocomplex, composed of cationic peptide and an ATP-binding aptamer-incorporated DNA scaffold with intercalated doxorubicin	A549 spheroids	Nanocomplexes caused a complete disruption of the spheroids at 24 h after treatment, whereas free doxorubicin at the same dose caused an insignificant cytotoxic effect	(104)
Transferrin-conjugated doxorubicin-loaded lipid-coated PLGA nanoparticles	A549 spheroids	Targeted nanoparticles exhibited threefold higher spheroid growth inhibition compared with non-targeted counterparts	(105)
Chitosan–cholesterol micelles loaded with curcumin	A549 spheroids	Superior penetration and complete inhibition of spheroid growth after treatment with nanoparticles in comparison with free curcumin	(106)
Docetaxel-loaded PEG-PLGA redox-responsive nanoparticles	A549 spheroids	The particles have shown enhanced penetration into the spheroids in comparison with non-redox-responsive counterparts	(107)
PEG-PCL polymeric nanoparticles conjugated with cell-penetrating peptide RLW and loaded with docetaxel	A549 spheroids	RLW-decorated nanoparticles abrogated spheroid growth and caused 20% shrinkage, whereas unmodified nanoparticles caused only spheroid growth inhibition	(108)

## Analysis of Drug Penetration Using NSCLC Spheroid Models

The efficacy of cancer treatment is strongly limited by the poor uptake and hindered penetration of anticancer drugs through the tumor interstitium. It should be noted that this problem is common for nanomedicines (109) and small-molecule drugs (110). There are multiple factors which impair the tumor uptake of anticancer drugs, including insufficient blood supply of a tumor, lack of lymphatic drainage and elevated interstitial fluid pressure in tumor tissue, and accumulated solid stress due to high ECM and cell density (27). The spheroid model reproduces most of these pathophysiological features of tumors. For this reason, spheroids can be used for the analysis of drug penetration and evaluation of the approaches aiming at the improvement of spatial drug distribution in tumor tissue.

### Analysis of Small-Molecule Drug Penetration Into NSCLC Spheroids

Diffusion is the main driving force of drug penetration in tumor tissue (111). It means that interstitial drug penetration is size dependent. However, even small-molecule therapeutics experience diffusive hindrance in tumor tissue—for example, accumulation of doxorubicin in solid tumors was observed only in regions with high blood vessel density (112).

A pioneering study by Kerr et al. demonstrated the limited distribution of doxorubicin in a NSCLC spheroid model composed of L-DAN cells. The authors also found that hydrophobic 4′-deoxydoxorubicin exhibits a more uniform distribution, higher uptake by the spheroids and a stronger cytotoxic effect than doxorubicin (78). In contrast to doxorubicin, which can be visualized due to excellent fluorescent properties, the analysis of tissue penetration of non-fluorescent small-molecule drugs is limited. However, measurement of their cytotoxicity on spheroids is appropriate for the evaluation of strategies which improve drug distribution in tumor tissue—for example, the application of sonoporation increased the anticancer efficacy of 5-fluorouracil, paclitaxel, and doxorubicin in A549-based spheroids for 1.2, 1.5, and eight times, respectively (113). Another study reports about the enhancement of paclitaxel-induced toxicity on an A549 spheroid model after a co-administration with the tumor-penetrating peptide iRGD. This peptide mediates tumor homing through binding to αv integrins, which are selectively expressed on various tumor cells. The tumor-penetrating properties of iRGD are mediated by a second sequence motif, which becomes exposed at the C-terminus of the peptide upon proteolytic processing of iRGD in tumors. Then, this motif binds to neuropilin-1 and activates a tanycytic transport pathway through the tumor tissue (114). It was shown that the co-administration of iRGD with paclitaxel led to a threefold increase of apoptotic cell number in A549 spheroids compared with paclitaxel alone (115). Therefore, the use of the mentioned strategies can improve the drug distribution and the efficacy of chemotherapy.

### Analysis of Nanomedicine Penetration Into NSCLC Spheroids

The diffusion of extravasated nanoparticles in tumor interstitium is strongly limited due to high ECM and cell density. However, they are able to penetrate tumor tissue due to the transcytosis pathway that was demonstrated using a spheroid model (116).

NSCLC spheroids are often used to compare the penetration of two or more types of nanoparticles aiming at the optimization of a nanoparticle design—for example, Varan et al. compared the penetration of different nanoformulations based on α- and β-cyclodextrins and loaded with erlotinib, a tyrosine kinase inhibitor. It was found that polycationic amphiphilic β-cyclodextrin-based nanocapsules of smaller size and positive surface charge exhibited the best A549 spheroid penetration capability than others (117). In another study, the authors compared the distribution of dendrimer-conjugated doxorubicin *versus* free drug in A549 and A549/NIH3T3 spheroids. It turned out that dendrimer conjugation enhances the penetration and cytotoxic efficacy of doxorubicin in ECM-expressing spheroids (28).

Another goal of nanoparticle penetration analysis on a spheroid model is testing of the strategies, which increases tumor tissue permeability for nanoparticles. In particular, the penetration of leukocyte-mimicking liposomes, enriched with transmembrane glycoproteins CD11a and CD11b, was studied in A549 spheroids. Cell adhesion molecules CD11a and CD11b mediate the interaction of leukocytes with the inflamed endothelium and the increase of its permeability. The authors have shown that the leukocyte-mimicking liposomes, loaded with doxorubicin, efficiently penetrated into A549 tumor spheroids and promoted a superior toxic effect compared with non-decorated liposomes (118). The authors of another study synthesized polymeric nanoparticles sensitive to pH/ROS/MMP-2 stimuli and loaded them with chlorine e6 and sorafenib. The nanoformulation was able to shed PEG corona and broke into smaller nanoparticles in response to stimuli that led to enhanced penetration into A549 spheroids in comparison with non-responsive nanoparticles (119).

It should be noted that there are multiple anti-desmoplastic strategies which potentiate the penetration of small molecules and nanomedicine and their therapeutic effect. Some of these approaches are under clinical trials (120). Once NSCLC tumors are characterized by excessive collagen deposition, a spheroid model seems to be a valuable tool for testing anti-fibrotic strategies.

## Future Prospects

The NSCLC spheroid model shares some physiological and molecular properties with tumors. For this reason, it can be used for numerous applications. Cell lines and tumor tissue are two main sources of cells for spheroid generation. The choice between cell line-based and organotypic spheroids depends on the goal of the study.

The use of well-established cancer cell lines (such as A549) and the control of spheroid composition enables us to carry out a reproducible head-to-head comparison of drug candidates which target certain or different cancer-specific signaling pathways. A large amount of information about NSCLC cell lines also helps to identify changes in cancer-specific gene expression during spheroid formation, which might play an important role in the development of chemoresistance and be a potential molecular target. Moreover, the reproducible properties of cell line-based spheroids make it feasible to tune the physicochemical properties of the drug candidates in terms of optimal penetration and cytotoxic properties in comparison with “gold-standard” anticancer drugs.

The key advantage of organotypic spheroids is genetic heterogeneity that enables us to consider this model for “personalized” medicine. Furthermore, patient-derived spheroids reproduce the cell composition and molecular expression profile of an original tumor. In fact, NSCLC is a highly heterogenous cancer, and there are no prognostic factors which would help us to choose the most appropriate drug combination for neoadjuvant or adjuvant chemotherapy. The choice of targeted therapy drugs is based on the mutational analysis of *EGFR*, *BRAF*, *ROS1*, and *ALK*, although there is no guarantee that a patient would benefit from it. A similar problem refers also to anti-PD-L1 and anti-CTLA-4 immunotherapies. Patient-derived spheroids ideally fulfil the need of experimental testing in all the mentioned therapeutic management options before the start of the patient treatment. Besides similarity with parent tumors, organotypic spheroids can be grown in a short-term period, and drug testing can be performed very fast.

At the same time, the predictive value of NSCLC spheroids is still not well realized. Despite an increasing number of studies, a large-scale correlation analysis between therapeutic outcome and drug testing on patient-derived spheroids has not been carried out yet. For the validation of drug testing results obtained using 3D multicellular spheroids, such parameters, as a type of NSCLC, mutational load, and cancer stage, should be taken into account. Some possible pitfalls cannot be excluded also—for example, spheroids from primary and metastatic tumors could give a different response to the same anticancer drugs because of their different clonal compositions. In this case, clear recommendations should be developed based on clinical data.

In-depth analysis of patient-derived spheroid properties might reveal the links between some of these properties and drug response. As a result, it would help to figure out relevant markers for the selection of certain drug combinations. We believe that further progress in this area will help to improve the therapeutic outcomes for NSCLC patients.

## Author Contributions

All authors listed have made a substantial, direct, and intellectual contribution to the work and approved it for publication.

## Funding

This work was financed by the Ministry of Science and Higher Education of the Russian Federation within the framework of state support for the creation and development of a World-Class Research Center “Digital Biodesign and Personalized Healthcare” (no. 075-15-2020-917).

## Conflict of Interest

The authors declare that the research was conducted in the absence of any commercial or financial relationships that could be construed as a potential conflict of interest.

## Publisher’s Note

All claims expressed in this article are solely those of the authors and do not necessarily represent those of their affiliated organizations, or those of the publisher, the editors and the reviewers. Any product that may be evaluated in this article, or claim that may be made by its manufacturer, is not guaranteed or endorsed by the publisher.

## References

[1] Lu T Yang X Huang Y Zhao M Li M Ma K. Trends in the Incidence, Treatment, and Survival of Patients With Lung Cancer in the Last Four Decades. Cancer Manag Res (2019) 11:943–53. doi: 10.2147/CMAR.S187317 PMC6345192 30718965

[2] Goto T Hirotsu Y Amemiya K Mochizuki H Omata M. Understanding Intratumor Heterogeneity and Evolution in NSCLC and Potential New Therapeutic Approach. Cancers (2018) 10:212. doi: 10.3390/cancers10070212 PMC6071014 29932159

[3] Testa U Castelli G Pelosi E. Lung Cancers: Molecular Characterization, Clonal Heterogeneity and Evolution, and Cancer Stem Cells. Cancers (2018) 10:248. doi: 10.3390/cancers10080248 PMC6116004 30060526

[4] Yamamoto A Iwata T. Expression Status of PD-L1 in NSCLC Correlates With Disease Extension. Eur Respir J (2018) 52:PA2804. doi: 10.1183/13993003.congress-2018.PA2804

[5] Lim SB Tan SJ Lim W-T Lim CT. An Extracellular Matrix-Related Prognostic and Predictive Indicator for Early-Stage Non-Small Cell Lung Cancer. Nat Commun (2017) 8:1734. doi: 10.1038/s41467-017-01430-6 29170406 PMC5700969

[6] Chu X Zhu C-C Liu H Wang J-C. Expression of Hypoxia-Inducible Factor Prolyl Hydroxylase 3 HIFPH3 in Human Non-Small Cell Lung Cancer (NSCLC) and Its Correlation With Prognosis. Asian Pacific J Cancer Prev (2014) 15:5819–23. doi: 10.7314/APJCP.2014.15.14.5819 25081707

[7] Monteiro CF Custódio CA Mano JF. Bioengineering a Humanized 3D Tri-Culture Osteosarcoma Model to Assess Tumor Invasiveness and Therapy Response. Acta Biomater (2021) 134:204–14. doi: 10.1016/j.actbio.2021.07.034 34303015

[8] Nath S Devi GR. Three-Dimensional Culture Systems in Cancer Research: Focus on Tumor Spheroid Model. Pharmacol Ther (2016) 163:94–108. doi: 10.1016/j.pharmthera.2016.03.013 27063403 PMC4961208

[9] Ryu N-E Lee S-H Park H. Spheroid Culture System Methods and Applications for Mesenchymal Stem Cells. Cells (2019) 8:1620. doi: 10.3390/cells8121620 PMC6953111 31842346

[10] Mrozik KM Blaschuk OW Cheong CM Zannettino ACW Vandyke K. N-Cadherin in Cancer Metastasis, its Emerging Role in Haematological Malignancies and Potential as a Therapeutic Target in Cancer. BMC Cancer (2018) 18:939. doi: 10.1186/s12885-018-4845-0 30285678 PMC6167798

[11] Hui L Zhang S Dong X Tian D Cui Z Qiu X. Prognostic Significance of Twist and N-Cadherin Expression in NSCLC. PloS One (2013) 8:e62171. doi: 10.1371/journal.pone.0062171 23626784 PMC3633889

[12] Huang Y-J Hsu S. Acquisition of Epithelial–Mesenchymal Transition and Cancer Stem-Like Phenotypes Within Chitosan-Hyaluronan Membrane-Derived 3D Tumor Spheroids. Biomaterials (2014) 35:10070–9. doi: 10.1016/j.biomaterials.2014.09.010 25282622

[13] Ziółkowska-Suchanek I. Mimicking Tumor Hypoxia in Non-Small Cell Lung Cancer Employing Three-Dimensional. In Vitro Models Cells (2021) 10:141. doi: 10.3390/cells10010141 33445709 PMC7828188

[14] Romero-Garcia S Lopez-Gonzalez JS Báez-Viveros JL Aguilar-Cazares D Prado-Garcia H. Tumor Cell Metabolism: An Integral View. Cancer Biol Ther (2011) 12:939–48. doi: 10.4161/cbt.12.11.18140 PMC3280912 22057267

[15] Sowa T Menju T Chen-Yoshikawa TF Takahashi K Nishikawa S Nakanishi T. Hypoxia-Inducible Factor 1 Promotes Chemoresistance of Lung Cancer by Inducing Carbonic Anhydrase IX Expression. Cancer Med (2017) 6:288–97. doi: 10.1002/cam4.991 PMC5269694 28028936

[16] Kim SJ Rabbani ZN Vollmer RT Schreiber E-G Oosterwijk E Dewhirst MW. Carbonic Anhydrase IX in Early-Stage Non-Small Cell Lung Cancer. Clin Cancer Res (2004) 10:7925–33. doi: 10.1158/1078-0432.CCR-04-0636 15585626

[17] Zheng G Peng C Jia X Gu Y Zhang Z Deng Y. ZEB1 Transcriptionally Regulated Carbonic Anhydrase 9 Mediates the Chemoresistance of Tongue Cancer. Via Maintaining Intracellular pH Mol Cancer (2015) 14:84. doi: 10.1186/s12943-015-0357-6 25890268 PMC4404088

[18] Swietach P Hulikova A Vaughan-Jones RD Harris AL. New Insights Into the Physiological Role of Carbonic Anhydrase IX in Tumour pH Regulation. Oncogene (2010) 29:6509–21. doi: 10.1038/onc.2010.455 20890298

[19] Dengler VL Galbraith MD Espinosa JM. Transcriptional Regulation by Hypoxia Inducible Factors. Crit Rev Biochem Mol Biol (2014) 49:1–15. doi: 10.3109/10409238.2013.838205 24099156 PMC4342852

[20] Bačić I Karlo R Zadro AŠ Zadro Z Skitarelić N Antabak A. Tumor Angiogenesis as an Important Prognostic Factor in Advanced Non-Small Cell Lung Cancer (Stage IIIA). Oncol Lett (2018) 15:2335–9. doi: 10.3892/ol.2017.7576 PMC5777107 29434942

[21] Liang H Wang M. Prospect of Immunotherapy Combined With Anti-Angiogenic Agents in Patients With Advanced Non-Small Cell Lung Cancer. Cancer Manag Res (2019) 11:7707–19. doi: 10.2147/CMAR.S212238 PMC6699593 31616186

[22] Arai K Eguchi T Rahman MM Sakamoto R Masuda N Nakatsura T. A Novel High-Throughput 3d Screening System for EMT Inhibitors: A Pilot Screening Discovered the EMT Inhibitory Activity of CDK2 Inhibitor Su9516. PloS One (2016) 11:e0162394. doi: 10.1371/journal.pone.0162394 27622654 PMC5021355

[23] Amann A Zwierzina M Koeck S Gamerith G Pechriggl E Huber JM. Development of a 3D Angiogenesis Model to Study Tumour – Endothelial Cell Interactions and the Effects of Anti-Angiogenic Drugs. Sci Rep (2017) 7:2963. doi: 10.1038/s41598-017-03010-6 28592821 PMC5462801

[24] Bremnes RM Dønnem T Al-Saad S Al-Shibli K Andersen S Sirera R. The Role of Tumor Stroma in Cancer Progression and Prognosis: Emphasis on Carcinoma-Associated Fibroblasts and Non-Small Cell Lung Cancer. J Thorac Oncol (2011) 6:209–17. doi: 10.1097/JTO.0b013e3181f8a1bd 21107292

[25] Liu T Zhou L Li D Andl T Zhang Y. Cancer-Associated Fibroblasts Build and Secure the Tumor Microenvironment. Front Cell Dev Biol (2019) 0:60. doi: 10.3389/fcell.2019.00060 PMC6492564 31106200

[26] Parker AL Cox TR. The Role of the ECM in Lung Cancer Dormancy and Outgrowth. Front Oncol (2020) 0:1766. doi: 10.3389/fonc.2020.01766 PMC7516130 33014869

[27] Durymanov MO Rosenkranz AA Sobolev AS. Current Approaches for Improving Intratumoral Accumulation and Distribution of Nanomedicines. Theranostics (2015) 5:1007–20. doi: 10.7150/thno.11742 PMC4493538 26155316

[28] Almuqbil RM Heyder RS Bielski ER Durymanov M Reineke JJ da Rocha SRP. Dendrimer Conjugation Enhances Tumor Penetration and Efficacy of Doxorubicin in Extracellular Matrix-Expressing 3d Lung Cancer Models. Mol Pharm (2020) 17:1648–62. doi: 10.1021/acs.molpharmaceut.0c00083 32227969

[29] Yie S Yang H Ye S Li K Dong D Lin X. Expression of Human Leucocyte Antigen G (HLA-G) is Associated With Prognosis in Non-Small Cell Lung Cancer. Lung Cancer (2007) 58:267–74. doi: 10.1016/j.lungcan.2007.06.011 17673327

[30] Bodor JN Boumber Y Borghaei H. Biomarkers for Immune Checkpoint Inhibition in Non–Small Cell Lung Cancer (NSCLC). Cancer (2020) 126:260–70. doi: 10.1002/cncr.32468 PMC7372560 31691957

[31] Wang X Wang Y Zhou Q Peng M Zhang J Chen M. Immunomodulatory Effect of Lentinan on Aberrant T Subsets and Cytokines Profile in Non-Small Cell Lung Cancer Patients. Pathol Oncol Res (2020) 26:499–505. doi: 10.1007/s12253-018-0545-y 30460541

[32] Mandarano M Bellezza G Belladonna ML Van den Eynde BJ Chiari R Vannucci J. Assessment of TILs, IDO-1, and PD-L1 in Resected Non-Small Cell Lung Cancer: An Immunohistochemical Study With Clinicopathological and Prognostic Implications. Virchows Arch (2019) 474:159–68. doi: 10.1007/s00428-018-2483-1 30448912

[33] Duan M-C Han W Jin P-W Wei Y-P Wei Q Zhang L-M. Disturbed Th17/Treg Balance in Patients With Non-Small Cell Lung Cancer. Inflammation (2015) 38:2156–65. doi: 10.1007/s10753-015-0198-x 26077695

[34] Yao Z Zhang J Zhang B Liang G Chen X Yao F. Imatinib Prevents Lung Cancer Metastasis by Inhibiting M2-Like Polarization of Macrophages. Pharmacol Res (2018) 133:121–31. doi: 10.1016/j.phrs.2018.05.002 29730267

[35] Rebelo SP Pinto C Martins TR Harrer N Estrada MF Loza-Alvarez P. 3D-3-Culture: A Tool to Unveil Macrophage Plasticity in the Tumour Microenvironment. Biomaterials (2018) 163:185–97. doi: 10.1016/j.biomaterials.2018.02.030 29477032

[36] Evans L Milward K Attanoos R Clayton A Errington R Tabi Z. Macrophage Plasticity and Function in the Lung Tumour Microenvironment Revealed in 3D Heterotypic Spheroid and Explant Models. Biomedicines (2021) 9:302. doi: 10.3390/biomedicines9030302 33804204 PMC7999110

[37] Aref AR Campisi M Ivanova E Portell A Larios D Piel BP. 3D Microfluidic *Ex Vivo* Culture of Organotypic Tumor Spheroids to Model Immune Checkpoint Blockade. Lab Chip (2018) 18:3129–43. doi: 10.1039/C8LC00322J PMC6274590 30183789

[38] Banda M McKim KL Myers MB Inoue M Parsons BL. Outgrowth of Erlotinib-Resistant Subpopulations Recapitulated in Patient-Derived Lung Tumor Spheroids and Organoids. PloS One (2020) 15:e0238862. doi: 10.1371/journal.pone.0238862 32898185 PMC7478813

[39] Abdul Satar N Ismail MN Yahaya BH. Synergistic Roles of Curcumin in Sensitising the Cisplatin Effect on a Cancer Stem Cell-Like Population Derived From Non-Small Cell Lung Cancer Cell Lines. Molecules (2021) 26:1056. doi: 10.3390/molecules26041056 33670440 PMC7922800

[40] Wang Y Zhou Y Tao F Chai S Xu X Yang Y. N-Myc Downstream Regulated Gene 1(NDRG1) Promotes the Stem-Like Properties of Lung Cancer Cells Through Stabilized C-Myc. Cancer Lett (2017) 401:53–62. doi: 10.1016/j.canlet.2017.04.031 28456659

[41] Zhao C Setrerrahmane S Xu H. Enrichment and Characterization of Cancer Stem Cells From a Human Non-Small Cell Lung Cancer Cell Line. Oncol Rep (2015) 34:2126–32. doi: 10.3892/or.2015.4163 26239272

[42] Leung EL-H Fiscus RR Tung JW Tin VP-C Cheng LC Sihoe AD-L. Non-Small Cell Lung Cancer Cells Expressing CD44 are Enriched for Stem Cell-Like Properties. PloS One (2010) 5:e14062. doi: 10.1371/journal.pone.0014062 21124918 PMC2988826

[43] Surapaneni SK Nottingham E Mondal A Patel N Arthur P Gebeyehu A. Telmisartan Facilitates the Anticancer Effects of CARP-1 Functional Mimetic and Sorafenib in Rociletinib Resistant Non-Small Cell Lung Cancer. Anticancer Res (2021) 41:4215–28. doi: 10.21873/anticanres.15226 PMC8691118 34475041

[44] Tan Q Lin S Zeng Y Yao M Liu K Yuan H. Ginsenoside Rg3 Attenuates the Osimertinib Resistance by Reducing the Stemness of Non-Small Cell Lung Cancer Cells. Environ Toxicol (2020) 35:643–51. doi: 10.1002/tox.22899 31916386

[45] Chen Q Luo J Wu C Lu H Cai S Bao C. The miRNA-149-5p/MyD88 Axis is Responsible for Ursolic Acid-Mediated Attenuation of the Stemness and Chemoresistance of Non-Small Cell Lung Cancer Cells. Environ Toxicol (2020) 35:561–9. doi: 10.1002/tox.22891 31855318

[46] Zhao Y Zheng R Chen J Ning D. CircRNA CDR1as/miR-641/HOXA9 Pathway Regulated Stemness Contributes to Cisplatin Resistance in Non-Small Cell Lung Cancer (NSCLC). Cancer Cell Int (2020) 20:289. doi: 10.1186/s12935-020-01390-w 32655321 PMC7339514

[47] Ni Y-L Hsieh C-H Kim S-H Wang J-P Su C-L Yao C-F. A Potent Indolylquinoline Alleviates Growth of Human Lung Cancer Cell Tumorspheres. Apoptosis (2017) 22:1235–45. doi: 10.1007/s10495-017-1401-3 28741092

[48] Herreros-Pomares A de-Maya-Girones JD Calabuig-Fariñas S Lucas R Martínez A Pardo-Sánchez JM. Lung Tumorspheres Reveal Cancer Stem Cell-Like Properties and a Score With Prognostic Impact in Resected Non-Small-Cell Lung Cancer. Cell Death Dis (2019) 10:660. doi: 10.1038/s41419-019-1898-1 31506430 PMC6737160

[49] Chevallier M Borgeaud M Addeo A Friedlaender A. Oncogenic Driver Mutations in Non-Small Cell Lung Cancer: Past, Present and Future. World J Clin Oncol (2021) 12:217–37. doi: 10.5306/wjco.v12.i4.217 PMC8085514 33959476

[50] Tripathi SK Biswal BK. SOX9 Promotes Epidermal Growth Factor Receptor-Tyrosine Kinase Inhibitor Resistance *via* Targeting β-Catenin and Epithelial to Mesenchymal Transition in Lung Cancer. Life Sci (2021) 277:119608. doi: 10.1016/j.lfs.2021.119608 33989664

[51] Murtuza A Bulbul A Shen JP Keshavarzian P Woodward BD Lopez-Diaz FJ. Novel Third-Generation EGFR Tyrosine Kinase Inhibitors and Strategies to Overcome Therapeutic Resistance in Lung Cancer. Cancer Res (2019) 79:689–98. doi: 10.1158/0008-5472.CAN-18-1281 30718357

[52] Mok TS Wu Y-L Ahn M-J Garassino MC Kim HR Ramalingam SS. Osimertinib or Platinum–Pemetrexed in EGFR T790M–Positive Lung Cancer. N Engl J Med (2017) 376:629–40. doi: 10.1056/NEJMoa1612674 PMC6762027 27959700

[53] Theard PL Sheffels E Sealover NE Linke AJ Pratico DJ Kortum RL. Marked Synergy by Vertical Inhibition of EGFR Signaling in NSCLC Spheroids Shows SOS1 is a Therapeutic Target in EGFR-Mutated Cancer. Elife (2020) 9:e58204. doi: 10.7554/eLife.58204 32897190 PMC7478890

[54] Canon J Rex K Saiki AY Mohr C Cooke K Bagal D. The Clinical KRAS(G12C) Inhibitor AMG 510 Drives Anti-Tumour Immunity. Nature (2019) 575:217–23. doi: 10.1038/s41586-019-1694-1 31666701

[55] Phiboonchaiyanan PP Puthongking P Chawjarean V Harikarnpakdee S Sukprasansap M Chanvorachote P. Melatonin and its Derivative Disrupt Cancer Stem-Like Phenotypes of Lung Cancer Cells *via* AKT Downregulation. Clin Exp Pharmacol Physiol (2021) 48:1712–23. doi: 10.1111/1440-1681.13572 34396568

[56] Yong X Wang P Jiang T Yu W Shang Y Han Y. Fibroblasts Weaken the Anti-Tumor Effect of Gefitinib on Co-Cultured Non-Small Cell Lung Cancer Cells. Chin Med J (Engl) (2014) 127:2091–6. doi: 10.3760/cma.j.issn.0366-6999.20133050 24890158

[57] Shi J Feng J Xie J Mei Z Shi T Wang S. Targeted Blockade of TGF-β and IL-6/JAK2/STAT3 Pathways Inhibits Lung Cancer Growth Promoted by Bone Marrow-Derived Myofibroblasts. Sci Rep (2017) 7:8660. doi: 10.1038/s41598-017-09020-8 28819126 PMC5561133

[58] Shintani Y Fujiwara A Kimura T Kawamura T Funaki S Minami M. IL-6 Secreted From Cancer-Associated Fibroblasts Mediates Chemoresistance in NSCLC by Increasing Epithelial-Mesenchymal Transition Signaling. J Thorac Oncol (2016) 11:1482–92. doi: 10.1016/j.jtho.2016.05.025 27287412

[59] Abulaiti A Shintani Y Funaki S Nakagiri T Inoue M Sawabata N. Interaction Between Non-Small-Cell Lung Cancer Cells and Fibroblasts *via* Enhancement of TGF-β Signaling by IL-6. Lung Cancer (2013) 82:204–13. doi: 10.1016/j.lungcan.2013.08.008 24011634

[60] Chen Y Zhang F Tsai Y Yang X Yang L Duan S. IL-6 Signaling Promotes DNA Repair and Prevents Apoptosis in CD133+ Stem-Like Cells of Lung Cancer After Radiation. Radiat Oncol (2015) 10:227. doi: 10.1186/s13014-015-0534-1 26572130 PMC4647293

[61] Liu C-C Lin J-H Hsu T-W Su K Li AF-Y Hsu H-S. IL-6 Enriched Lung Cancer Stem-Like Cell Population by Inhibition of Cell Cycle Regulators *via* DNMT1 Upregulation. Int J Cancer (2015) 136:547–59. doi: 10.1002/ijc.29033 24947242

[62] Galland S Martin P Fregni G Letovanec I Stamenkovic I. Attenuation of the Pro-Inflammatory Signature of Lung Cancer-Derived Mesenchymal Stromal Cells by Statins. Cancer Lett (2020) 484:50–64. doi: 10.1016/j.canlet.2020.05.005 32418888

[63] Kim M Mun H Sung CO Cho EJ Jeon H-J Chun S-M. Patient-Derived Lung Cancer Organoids as *In Vitro* Cancer Models for Therapeutic Screening. Nat Commun (2019) 10:3991. doi: 10.1038/s41467-019-11867-6 31488816 PMC6728380

[64] Lee M Song Y Choi I Lee S-Y Kim S Kim S-H. Expression of HYOU1 *via* Reciprocal Crosstalk Between NSCLC Cells and HUVECs Control Cancer Progression and Chemoresistance in Tumor Spheroids. Mol Cells (2021) 44:50–62. doi: 10.14348/molcells.2020.0212 33455947 PMC7854178

[65] Kim S-H Song Y Seo HR. GSK-3β Regulates the Endothelial-to-Mesenchymal Transition *via* Reciprocal Crosstalk Between NSCLC Cells and HUVECs in Multicellular Tumor Spheroid Models. J Exp Clin Cancer Res (2019) 38:46. doi: 10.1186/s13046-019-1050-1 30709379 PMC6359813

[66] Busse A Letsch A Fusi A Nonnenmacher A Stather D Ochsenreither S. Characterization of Small Spheres Derived From Various Solid Tumor Cell Lines: Are They Suitable Targets for T Cells? Clin Exp Metastasis (2013) 30:781–91. doi: 10.1007/s10585-013-9578-5 23519726

[67] Della Corte CM Barra G Ciaramella V Di Liello R Vicidomini G Zappavigna S. Antitumor Activity of Dual Blockade of PD-L1 and MEK in NSCLC Patients Derived Three-Dimensional Spheroid Cultures. J Exp Clin Cancer Res (2019) 38:253. doi: 10.1186/s13046-019-1257-1 31196138 PMC6567578

[68] Gaudreau P-O Lee JJ Heymach JV Gibbons DL. Phase I/II Trial of Immunotherapy With Durvalumab and Tremelimumab With Continuous or Intermittent MEK Inhibitor Selumetinib in NSCLC: Early Trial Report. Clin Lung Cancer (2020) 21:384–8. doi: 10.1016/j.cllc.2020.02.019 PMC7656492 32299768

[69] Sherman H Gitschier HJ. Rossi AE. A Novel Three-Dimensional Immune Oncology Model for High-Throughput Testing of Tumoricidal Activity. Front Immunol (2018) 9:857. doi: 10.3389/fimmu.2018.00857 29740450 PMC5924962

[70] Varudkar N Oyer JL Copik A Parks GD. Oncolytic Parainfluenza Virus Combines With NK Cells to Mediate Killing of Infected and Non-Infected Lung Cancer Cells Within 3D Spheroids: Role of Type I and Type III Interferon Signaling. J Immunother Cancer (2021) 9:e002373. doi: 10.1136/jitc-2021-002373 34172515 PMC8237729

[71] Tazzyman S Barry ST Ashton S Wood P Blakey D Lewis CE. Inhibition of Neutrophil Infiltration Into A549 Lung Tumors *In Vitro* and *In Vivo* Using a CXCR2-Specific Antagonist is Associated With Reduced Tumor Growth. Int J Cancer (2011) 129:847–58. doi: 10.1002/ijc.25987 21328342

[72] Artal Cortés Á Calera Urquizu L Hernando Cubero J. Adjuvant Chemotherapy in Non-Small Cell Lung Cancer: State-of-the-Art. Transl Lung Cancer Res (2015) 4:191–7. doi: 10.3978/j.issn.2218-6751.2014.06.01 PMC4384209 25870801

[73] Green MR Andrews M Leff R Willey J Allegra C Denes A. Adjuvant Therapy Choices in Patients With Resected Non-Small-Cell Lung Cancer: Correlation of Doctors’ Treatment Plans and Relevant Phase III Trial Data. J Oncol Pract (2005) 1:37–42. doi: 10.1200/jop.2005.1.2.37 20871677 PMC2793579

[74] Kosmidis P Mylonakis N Nicolaides C Kalophonos C Samantas E Boukovinas J. Paclitaxel Plus Carboplatin Versus Gemcitabine Plus Paclitaxel in Advanced Non–Small-Cell Lung Cancer: A Phase III Randomized Trial. JCO (2002) 20:3578–85. doi: 10.1200/JCO.2002.12.112 12202657

[75] Chevalier TL. Adjuvant Chemotherapy for Resectable Non-Small-Cell Lung Cancer: Where is it Going? Ann Oncol (2010) 21:vii196–8. doi: 10.1093/annonc/mdq376 20943614

[76] Huber JM Amann A Koeck S Lorenz E Kelm JM Obexer P. Evaluation of Assays for Drug Efficacy in a Three-Dimensional Model of the Lung. J Cancer Res Clin Oncol (2016) 142:1955–66. doi: 10.1007/s00432-016-2198-0 PMC4978763 27424189

[77] Barrera-Rodríguez R Fuentes JM. Multidrug Resistance Characterization in Multicellular Tumour Spheroids From Two Human Lung Cancer Cell Lines. Cancer Cell Int (2015) 15:47. doi: 10.1186/s12935-015-0200-6 26221079 PMC4517505

[78] Kerr DJ Wheldon TE Hydns S Kaye SB. Cytotoxic Drug Penetration Studies in Multicellular Tumour Spheroids. Xenobiotica (1988) 18:641–8. doi: 10.3109/00498258809041702 3166552

[79] Millard M Yakavets I Zorin V Kulmukhamedova A Marchal S Bezdetnaya L. Drug Delivery to Solid Tumors: The Predictive Value of the Multicellular Tumor Spheroid Model for Nanomedicine Screening. Int J Nanomed (2017) 12:7993–8007. doi: 10.2147/IJN.S146927 PMC5673046 29184400

[80] Maruhashi R Akizuki R Sato T Matsunaga T Endo S Yamaguchi M. Elevation of Sensitivity to Anticancer Agents of Human Lung Adenocarcinoma A549 Cells by Knockdown of Claudin-2 Expression in Monolayer and Spheroid Culture Models. Biochim Biophys Acta Mol Cell Res (2018) 1865:470–9. doi: 10.1016/j.bbamcr.2017.12.005 29247669

[81] Kamer I Bab-Dinitz E Zadok O Ofek E Gottfried T Daniel-Meshulam I. Immunotherapy Response Modeling by *Ex-Vivo* Organ Culture for Lung Cancer. Cancer Immunol Immunother (2021) 70:2223–34. doi: 10.1007/s00262-020-02828-w PMC10992805 33484295

[82] Zhang Z Wang H Ding Q Xing Y Xu Z Lu C. Establishment of Patient-Derived Tumor Spheroids for non-Small Cell Lung Cancer. PloS One (2018) 13:e0194016. doi: 10.1371/journal.pone.0194016 29543851 PMC5854348

[83] Ivanova E Kuraguchi M Xu M Portell AJ Taus L Diala I. Use of *Ex Vivo* Patient-Derived Tumor Organotypic Spheroids to Identify Combination Therapies for Her2 Mutant non–Small Cell Lung Cancer. Clin Cancer Res (2020) 26:2393–403. doi: 10.1158/1078-0432.CCR-19-1844 PMC7967092 32034078

[84] Di Liello R Ciaramella V Barra G Venditti M Della Corte CM Papaccio F. *Ex Vivo* Lung Cancer Spheroids Resemble Treatment Response of a Patient With NSCLC to Chemotherapy and Immunotherapy: Case Report and Translational Study. ESMO Open (2019) 4:e000536. doi: 10.1136/esmoopen-2019-000536 31555484 PMC6735672

[85] Berghmans T Dingemans A-M Hendriks LEL Cadranel J. Immunotherapy for Nonsmall Cell Lung Cancer: A New Therapeutic Algorithm. Eur Respir J (2020) 55:1901907. doi: 10.1183/13993003.01907-2019 32029641

[86] Gettinger S Horn L Jackman D Spigel D Antonia S Hellmann M. Five-Year Follow-Up of Nivolumab in Previously Treated Advanced Non-Small-Cell Lung Cancer: Results From the CA209-003 Study. J Clin Oncol (2018) 36:1675–84. doi: 10.1200/JCO.2017.77.0412 29570421

[87] Jenkins RW Aref AR Lizotte PH Ivanova E Stinson S Zhou CW. *Ex Vivo* Profiling of PD-1 Blockade Using Organotypic Tumor Spheroids. Cancer Discovery (2018) 8:196–215. doi: 10.1158/2159-8290.CD-17-0833 29101162 PMC5809290

[88] Jayson GC Kerbel R Ellis LM Harris AL. Antiangiogenic Therapy in Oncology: Current Status and Future Directions. Lancet (2016) 388:518–29. doi: 10.1016/S0140-6736(15)01088-0 26853587

[89] Tian W Cao C Shu L Wu F. Anti-Angiogenic Therapy in the Treatment of Non-Small Cell Lung Cancer. Onco Targets Ther (2020) 13:12113–29. doi: 10.2147/OTT.S276150 PMC7699985 33262610

[90] Munkhbaatar E Dietzen M Agrawal D Anton M Jesinghaus M Boxberg M. MCL-1 Gains Occur With High Frequency in Lung Adenocarcinoma and can be Targeted Therapeutically. Nat Commun (2020) 11:4527. doi: 10.1038/s41467-020-18372-1 32913197 PMC7484793

[91] Commander R Wei C Sharma A Mouw JK Burton LJ Summerbell E. Subpopulation Targeting of Pyruvate Dehydrogenase and GLUT1 Decouples Metabolic Heterogeneity During Collective Cancer Cell Invasion. Nat Commun (2020) 11:1533. doi: 10.1038/s41467-020-15219-7 32210228 PMC7093428

[92] Lee J-Y Talhi O Jang D Cerella C Gaigneaux A Kim K-W. Cytostatic Hydroxycoumarin OT52 Induces ER/Golgi Stress and STAT3 Inhibition Triggering non-Canonical Cell Death and Synergy With BH3 Mimetics in Lung Cancer. Cancer Lett (2018) 416:94–108. doi: 10.1016/j.canlet.2017.12.007 29247826

[93] Gao C Shen Y Jin F Miao Y Qiu X. Cancer Stem Cells in Small Cell Lung Cancer Cell Line H446: Higher Dependency on Oxidative Phosphorylation and Mitochondrial Substrate-Level Phosphorylation Than Non-Stem Cancer Cells. PloS One (2016) 11:e0154576. doi: 10.1371/journal.pone.0154576 27167619 PMC4863974

[94] Yang Y Xu H Huang W Ding M Xiao J Yang D. Targeting Lung Cancer Stem-Like Cells With TRAIL Gene Armed Oncolytic Adenovirus. J Cell Mol Med (2015) 19:915–23. doi: 10.1111/jcmm.12397 PMC4420595 25683371

[95] Kharbanda A Rajabi H Jin C Alam M Wong K-K Kufe D. MUC1-C Confers EMT and KRAS Independence in Mutant KRAS Lung Cancer Cells. Oncotarget (2014) 5:8893–905. doi: 10.18632/oncotarget.2360 PMC4253405 25245423

[96] Yeh C-T Wu ATH Chang PM-H Chen K-Y Yang C-N Yang S-C. Trifluoperazine, an Antipsychotic Agent, Inhibits Cancer Stem Cell Growth and Overcomes Drug Resistance of Lung Cancer. Am J Respir Crit Care Med (2012) 186:1180–8. doi: 10.1164/rccm.201207-1180OC 23024022

[97] Lautié E Russo O Ducrot P Boutin JA. Unraveling Plant Natural Chemical Diversity for Drug Discovery Purposes. Front Pharmacol (2020) 11:397. doi: 10.3389/fphar.2020.00397 32317969 PMC7154113

[98] Tripathi SK Rengasamy KRR Biswal BK. Plumbagin Engenders Apoptosis in Lung Cancer Cells *via* Caspase-9 Activation and Targeting Mitochondrial-Mediated ROS Induction. Arch Pharm Res (2020) 43:242–56. doi: 10.1007/s12272-020-01221-6 32034669

[99] Wang Y. Effects of Salinomycin on Cancer Stem Cell in Human Lung Adenocarcinoma A549 Cells. Med Chem (2011) 7:106–11. doi: 10.2174/157340611794859307 21222617

[100] Ilhan-Ayisigi E Ulucan F Saygili E Saglam-Metiner P Gulce-Iz S Yesil-Celiktas O. Nano-Vesicular Formulation of Propolis and Cytotoxic Effects in a 3D Spheroid Model of Lung Cancer. J Sci Food Agric (2020) 100:3525–35. doi: 10.1002/jsfa.10400 32239766

[101] Heng WS Cheah S-C. Identification of Phytochemical-Based β-Catenin Nuclear Localization Inhibitor in NSCLC: Differential Targeting Population From Member of Isothiocyanates. Molecules (2021) 26:399. doi: 10.3390/molecules26020399 PMC7828655 33451160

[102] Danhier F. To Exploit the Tumor Microenvironment: Since the EPR Effect Fails in the Clinic, What is the Future of Nanomedicine? J Control Release (2016) 244:108–21. doi: 10.1016/j.jconrel.2016.11.015 27871992

[103] Schütz CA Juillerat-Jeanneret L Mueller H Lynch I Riediker M. Therapeutic Nanoparticles in Clinics and Under Clinical Evaluation. Nanomedicine (2013) 8:449–67. doi: 10.2217/nnm.13.8 23477336

[104] Lu S Zhao F Zhang Q Chen P. Therapeutic Peptide Amphiphile as a Drug Carrier With ATP-Triggered Release for Synergistic Effect, Improved Therapeutic Index, and Penetration of 3D Cancer Cell Spheroids. Int J Mol Sci (2018) 19:2773. doi: 10.3390/ijms19092773 PMC6165277 30223518

[105] Guo Y Wang L Lv P Zhang P. Transferrin-Conjugated Doxorubicin-Loaded Lipid-Coated Nanoparticles for the Targeting and Therapy of Lung Cancer. Oncol Lett (2015) 9:1065–72. doi: 10.3892/ol.2014.2840 PMC4315058 25663858

[106] Muddineti OS Kumari P Ray E Ghosh B Biswas S. Curcumin-Loaded Chitosan–Cholesterol Micelles: Evaluation in Monolayers and 3D Cancer Spheroid Model. Nanomedicine (2017) 12:1435–53. doi: 10.2217/nnm-2017-0036 28573926

[107] Conte C Mastrotto F Taresco V Tchoryk A Quaglia F Stolnik S. Enhanced Uptake in 2D- and 3D- Lung Cancer Cell Models of Redox Responsive PEGylated Nanoparticles With Sensitivity to Reducing Extra- and Intracellular Environments. J Controlled Release (2018) 277:126–41. doi: 10.1016/j.jconrel.2018.03.011 29534890

[108] Gao H Zhang Q Yang Y Jiang X He Q. Tumor Homing Cell Penetrating Peptide Decorated Nanoparticles Used for Enhancing Tumor Targeting Delivery and Therapy. Int J Pharm (2015) 478:240–50. doi: 10.1016/j.ijpharm.2014.11.029 25448586

[109] Ding J Chen J Gao L Jiang Z Zhang Y Li M. Engineered Nanomedicines With Enhanced Tumor Penetration. Nano Today (2019) 29:100800. doi: 10.1016/j.nantod.2019.100800

[110] Marcucci F Corti A. How to Improve Exposure of Tumor Cells to Drugs—Promoter Drugs Increase Tumor Uptake and Penetration of Effector Drugs. Advanced Drug Deliv Rev (2012) 64:53–68. doi: 10.1016/j.addr.2011.09.007 21983328

[111] Chauhan VP Stylianopoulos T Boucher Y Jain RK. Delivery of Molecular and Nanoscale Medicine to Tumors: Transport Barriers and Strategies. Annu Rev Chem Biomol Eng (2011) 2:281–98. doi: 10.1146/annurev-chembioeng-061010-114300 22432620

[112] Primeau AJ Rendon A Hedley D Lilge L Tannock IF. The Distribution of the Anticancer Drug Doxorubicin in Relation to Blood Vessels in Solid Tumors. Clin Cancer Res (2005) 11:8782–8. doi: 10.1158/1078-0432.CCR-05-1664 16361566

[113] Petrikaite V Paškevičiūtė M Raišutis R Sakalauskienė K. 14p - Application of Sonoporation to Increase Anticancer Drug Efficacy in 2D and 3D NSCLC Cell Cultures. Ann Oncol (2019) 30:v4–5. doi: 10.1093/annonc/mdz238.013

[114] Teesalu T Sugahara KN Ruoslahti E. Tumor-Penetrating Peptides. Front Oncol (2013) 3:216. doi: 10.3389/fonc.2013.00216 23986882 PMC3753659

[115] Gupta SK Torrico Guzmán EA Meenach SA. Coadministration of a Tumor-Penetrating Peptide Improves the Therapeutic Efficacy of Paclitaxel in a Novel Air-Grown Lung Cancer 3D Spheroid Model. Int J Cancer (2017) 141:2143–53. doi: 10.1002/ijc.30913 PMC5769484 28771722

[116] Durymanov M Kroll C Permyakova A Reineke J. Role of Endocytosis in Nanoparticle Penetration of 3D Pancreatic Cancer Spheroids. Mol Pharm (2019) 19:2773. doi: 10.1021/acs.molpharmaceut.8b01078 30707590

[117] Varan G Akkın S Demirtürk N Benito JM Bilensoy E. Erlotinib Entrapped in Cholesterol-Depleting Cyclodextrin Nanoparticles Shows Improved Antitumoral Efficacy in 3D Spheroid Tumors of the Lung and the Liver. J Drug Targeting (2021) 29:439–53. doi: 10.1080/1061186X.2020.1853743 33210947

[118] Fukuta T Yoshimi S Kogure K. Leukocyte-Mimetic Liposomes Penetrate Into Tumor Spheroids and Suppress Spheroid Growth by Encapsulated Doxorubicin. J Pharm Sci (2021) 110:1701–9. doi: 10.1016/j.xphs.2020.10.049 33129835

[119] Shu M Tang J Chen L Zeng Q Li C Xiao S. Tumor Microenvironment Triple-Responsive Nanoparticles Enable Enhanced Tumor Penetration and Synergetic Chemo-Photodynamic Therapy. Biomaterials (2021) 268:120574. doi: 10.1016/j.biomaterials.2020.120574 33271451

[120] Nandi T Pradyuth S Singh AK Chitkara D Mittal A. Therapeutic Agents for Targeting Desmoplasia: Current Status and Emerging Trends. Drug Discov Today (2020) 478(2015):240–50. doi: 10.1016/j.drudis.2020.09.008 32947044

